# A Ku-Band 13 W GaN HEMT Power Amplifier MMIC with a Coupled-Line Interstage Stabilization Technique for Radar Sensor Systems

**DOI:** 10.3390/s26082508

**Published:** 2026-04-18

**Authors:** Jihoon Kim

**Affiliations:** School of Electronic Engineering, Kyonggi University, Suwon 16227, Republic of Korea; j7h7@kgu.ac.kr; Tel.: +82-31-249-9803

**Keywords:** Ku-band, GaN HEMT, MMIC, power amplifier, coupled line, stability

## Abstract

This paper presents a 13 W Ku-band GaN HEMT MMIC power amplifier employing a coupled-line interstage stabilization technique for radar sensor front-end applications. High-efficiency and stable power amplification in the Ku-band is essential for radar sensing systems, where low-frequency instability and process sensitivity often limit multistage GaN amplifier performance. To address these challenges, a coupled-line interstage network is introduced instead of conventional series capacitors and parallel RC stabilization circuits. The proposed structure effectively suppresses low-frequency gain while maintaining RF performance and improving robustness against process variations due to its planar transmission-line implementation. The two-stage power amplifier was fabricated using a 0.25 μm commercial GaN HEMT MMIC process. For compact implementation, the coupled-line structure was realized in a meandered layout and verified through full electromagnetic simulations. Measured small-signal results show a gain (S21) of 18.6–21.6 dB, with input and output return losses (S11 and S22) of −3.3 to −10.2 dB and −4.4 to −7.2 dB, respectively, over 13.5–16 GHz. Large-signal measurements demonstrate a saturated output power of 40.7–41.5 dBm and a power-added efficiency of 21.3–28.1% across the same frequency range. The fabricated MMIC achieved stable operation without oscillation, validating the effectiveness of the proposed coupled-line stabilization approach for Ku-band radar sensor systems.

## 1. Introduction

**Figure 1:**
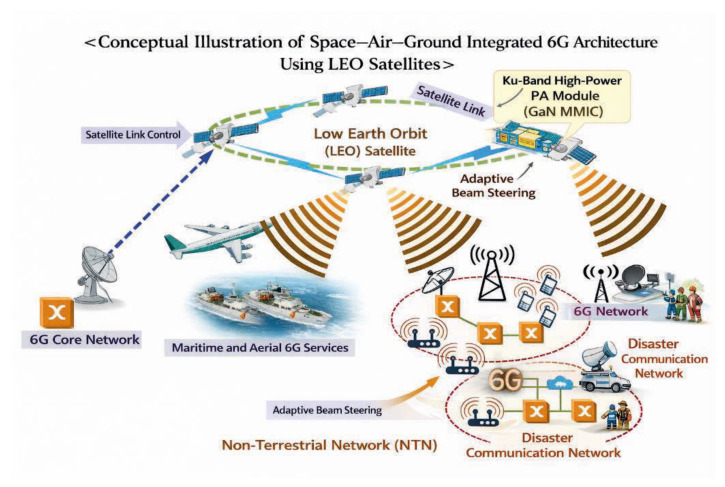
Conceptual illustration of integrated radar sensor communication through satellite–terrestrial networks.

Recent advances in radar sensors and satellite communication systems have increased the demand for reliable wireless links in diverse environments. In particular, sensor data collected from distributed platforms such as maritime, aviation, and disaster monitoring systems often require long-distance transmission through integrated terrestrial and satellite communication links. A conceptual illustration of such a sensor-based communication framework is shown in Figure 1, where data from radar sensors and wireless sensor nodes are transmitted via combined terrestrial and satellite networks. To support this framework, RF transmitter front-ends capable of stable operation and high output power are essential.

In these systems, high-power amplifiers (PAs) operating in the Ku-band play a critical role in achieving long-distance communication and maintaining a high signal-to-noise ratio (SNR). To meet these requirements, GaN HEMT technologies, which offer high breakdown voltage and high power density, have been widely adopted. Consequently, various Ku-band GaN MMIC PAs have been actively studied and reported [1,2,3].

To achieve high output power, PAs are typically implemented using multistage architectures. However, multistage PAs are inherently prone to low-frequency oscillations, making stabilization a critical design challenge. Conventional approaches employ RC-based stabilization networks or interstage structures incorporating series capacitors [4,5]. Although effective in improving stability, these techniques introduce additional loss, which degrades gain and high-frequency performance, and may increase sensitivity to design parameters. This issue becomes more critical in monolithic microwave integrated circuits (MMICs), where post-fabrication tuning is practically impossible. Insufficient stability may result in oscillations that degrade performance or even prevent proper measurement. Therefore, it is essential to incorporate effective stabilization techniques at the initial design stage.

To address these challenges, this paper proposes an interstage stabilization structure based on coupled transmission lines. The proposed structure suppresses low-frequency gain while maintaining high-frequency performance, thereby improving the stability of multistage PAs without introducing significant additional loss. Furthermore, a meander-type layout is employed to reduce chip area while preserving the desired electrical characteristics. Unlike conventional approaches that rely on additional RC networks or feedback circuits, the proposed structure simultaneously achieves impedance matching and low-frequency gain suppression. As a result, the proposed Ku-band GaN MMIC PA achieves oscillation-free operation without additional stabilization circuits while maintaining high output power and efficiency.

In this work, the proposed interstage structure is applied to the design of a two-stage Ku-band GaN HEMT MMIC PA. The operating principle is analyzed, and its performance is verified through electromagnetic (EM) simulations and measurements.

The remainder of this paper is organized as follows. Section 2 describes the operating principle of the proposed coupled-line structure. Section 3 presents the PA design. Section 4 provides measurement results and comparison with prior works. Finally, Section 5 concludes the paper.

## 2. Interstage Coupled-Line Design

### 2.1. Interstage Network for Stability

**Figure 2:**
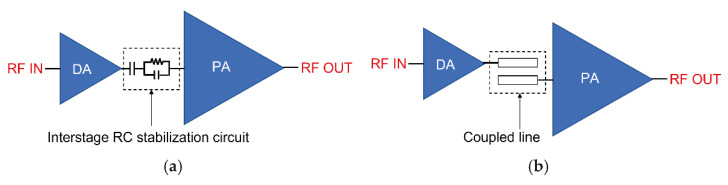
Conventional and proposed interstage networks: (**a**) RC-based structure and (**b**) coupled-line structure.

In conventional PA architectures, as shown in Figure 2a, a series capacitor and an RC stabilization network are typically inserted in the interstage network between the driver amplifier (DA) and the PA to reduce the low-frequency gain. This approach has the advantage of effectively suppressing low-frequency gain and improving stability. However, it still has certain limitations because additional stabilization components must be connected to the gate of each transistor. As a result, the inclusion of extra passive elements increases circuit complexity and introduces additional losses [4,5].

In contrast, the structure proposed in this paper applies an interstage network based on a coupled transmission line, as illustrated in Figure 2b, between the driver amplifier and the PA. Coupled transmission lines can exhibit frequency-dependent transmission characteristics by utilizing the electromagnetic coupling between adjacent transmission lines. Through this mechanism, signal transmission in the low-frequency region can be effectively suppressed without employing additional resistive components.

Furthermore, since the proposed structure is implemented using a transmission-line-based planar configuration, it can be relatively easily realized in an MMIC layout and does not introduce additional resistive losses. Therefore, the proposed coupled-line-based interstage structure can serve as an effective design approach for improving the stability of multistage GaN MMIC PAs while minimizing degradation in high-frequency performance.

### 2.2. Interstage Coupled-Line Structure

The coupled line used in the proposed interstage network consists of two transmission lines placed in close proximity, allowing electromagnetic coupling between them. Such structures are widely used in microwave circuits for various applications, including filters, directional couplers, and impedance transformers. By utilizing the electrical coupling between the two transmission lines, the structure can realize desired transmission characteristics at specific frequencies [6].

**Figure 3:**
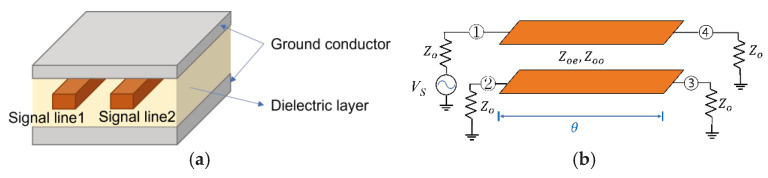
Coupled-line structure used in the proposed interstage network: (**a**) physical stripline configuration and (**b**) equivalent transmission-line model with electrical length θ.

Figure 3a shows the stripline-based coupled-line structure considered in this study. Two signal transmission lines (signal line 1 and signal line 2) are arranged in parallel within the dielectric substrate, while ground conductors are formed on the upper and lower sides. This stripline configuration confines most of the electromagnetic fields within the dielectric medium, thereby minimizing the influence of the external environment and providing stable transmission characteristics. When the two transmission lines are placed sufficiently close to each other, they no longer operate independently but instead behave as a coupled transmission line structure.

Figure 3b illustrates an equivalent circuit model of the coupled-line structure shown in Figure 3a based on transmission-line theory [6]. The electromagnetic coupling between the two transmission lines is determined by the electrical length θ of the transmission line and the modal characteristic impedances. In general, such coupled transmission lines can be analyzed using two propagation modes: the even mode and the odd mode [6]. In the even mode, voltages with the same phase are applied to the two transmission lines, whereas in the odd mode, voltages with opposite phases are applied. Each mode exhibits different characteristic impedances, which are defined as the even-mode impedance ($Z_{oe}$) and the odd-mode impedance ($Z_{oo}$), respectively.

**Figure 4:**
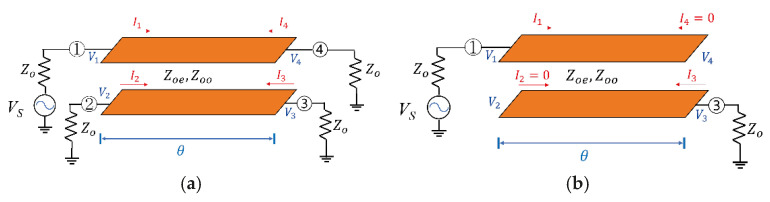
Equivalent circuit models of the coupled-line structure: (**a**) four-port coupled-line representation and (**b**) simplified two-port model used for theoretical analysis.

To analyze the transmission characteristics of the coupled line, an equivalent circuit model was constructed as shown in Figure 4. Figure 4a illustrates a model in which the coupled line is represented as a four-port network. Each transmission line is terminated with a characteristic impedance ($Z_{o}$), while the electromagnetic coupling between the two lines is described by the $Z_{oe}$ and $Z_{oo}$, respectively. In addition, the length of the transmission line can be expressed in terms of the electrical length θ. In this case, the well-known four-port Z-parameters of the transmission line network can be expressed as follows [7,8]:

(1) $$\left[\begin{array}{c} V_{1}\\ V_{2}\\ V_{3}\\ V_{4} \end{array}\right]=\left[\begin{array}{c} Z_{11}\;Z_{12}\;Z_{13}\;Z_{14}\\ Z_{21}\;Z_{22}\;Z_{23}\;Z_{24}\\ Z_{31}\;Z_{32}\;Z_{33}\;Z_{34}\\ Z_{41}\;Z_{42}\;Z_{43}\;Z_{44} \end{array}\right]\left[\begin{array}{c} I_{1}\\ I_{2}\\ I_{3}\\ I_{4} \end{array}\right]$$

Each Z-parameter component constituting the matrix can be derived as follows.

(2) Z11=Z44=Zoecothγel1−ReRo+Zoocothγol1−RoRe

(3) Z12=Z21=Z34=Z43=ZoeRecothγel1−ReRo+ZooRocothγol1−RoRe

(4) Z13=Z31=Z24=Z42=ZoeRecschγel1−ReRo+ZooRocschγol1−RoRe

(5) Z14=Z41=Zoecschγel1−ReRo+Zoocschγol1−RoRe

(6) Z22=Z33=ZoeRe2cothγel1−ReRo+ZooRo2cothγol1−RoRe

(7) Z23=Z32=ZoeRe2cschγel1−ReRo+ZooRo2cschγol1−RoRe

where $\gamma_{e}$ and $\gamma_{o}$ are the propagation constants of even and odd modes; $R_{e}$ and $R_{o}$, the ratios of the voltages on the two lines for even and odd modes; l, the length of coupled lines.

For simplicity, assuming an ideal lossless condition where the coupled line is symmetric and the two transmission lines are embedded in the same dielectric medium, the parameters can be set as $R_{e}=1$, $R_{o}=-1$, and $\gamma_{e}l=\gamma_{o}l=j\theta$. In this case, Equations (2)–(7) above can be expressed as follows.

(8) $$Z_{11}=Z_{22}=Z_{33}=Z_{44}=\frac{Z_{oe}\coth j\beta l}{2}+\frac{Z_{oo}\coth j\beta l}{2}=-j\frac{\left(Z_{oe}+Z_{oo}\right)}{2}\cot\theta$$

(9) $$Z_{12}=Z_{21}=Z_{34}=Z_{43}=\frac{Z_{oe}\coth j\beta l}{2}-\frac{Z_{oo}R_{o}\coth j\beta l}{2}=-j\frac{\left(Z_{oe}-Z_{oo}\right)}{2}\cot\theta$$

(10) $$Z_{13}=Z_{31}=Z_{24}=Z_{42}=\frac{Z_{oe}\csc j\beta l}{2}-\frac{Z_{oo}\csc j\beta l}{2}=-j\frac{\left(Z_{oe}-Z_{oo}\right)}{2}\csc\theta$$

(11) $$Z_{14}=Z_{23}=Z_{32}=Z_{41}=\frac{Z_{oe}\csc j\beta l}{2}+\frac{Z_{oo}\csc j\beta l}{2}=-j\frac{\left(Z_{oe}+Z_{oo}\right)}{2}\csc\theta$$

Furthermore, when one of the ports of the coupled transmission lines is terminated with an open circuit, the coupled-line structure can be simplified and analyzed as illustrated in Figure 4b. Under this condition, no current flows through Port 2 and Port 4, resulting in the conditions $I_{2}=0$ and $I_{4}=0$, respectively. Therefore, each element of the Z-parameter matrix can be derived from the above equations and expressed as follows.

(12) $$\left[\begin{array}{c} V_{1}\\ V_{2}\\ V_{3}\\ V_{4} \end{array}\right]=\left[\begin{array}{c} Z_{11}\;Z_{12}\;Z_{13}\;Z_{14}\\ Z_{21}\;Z_{22}\;Z_{23}\;Z_{24}\\ Z_{31}\;Z_{32}\;Z_{33}\;Z_{34}\\ Z_{41}\;Z_{42}\;Z_{43}\;Z_{44} \end{array}\right]\left[\begin{array}{c} I_{1}\\ 0\\ I_{3}\\ 0 \end{array}\right]=\left[\begin{array}{c} Z_{11}I_{1}+Z_{13}I_{3}\\ Z_{21}I_{1}+Z_{23}I_{3}\\ Z_{31}I_{1}+Z_{33}I_{3}\\ Z_{41}I_{1}+Z_{43}I_{3} \end{array}\right]$$

Taking the two-port Z-parameter matrix formed by ports 1 and 3 from the matrix in (12) yields the following matrix.

(13) $$\left[\begin{array}{c} V_{1}^{\prime }\\ V_{2}^{\prime } \end{array}\right]=\left[\begin{array}{cc} Z_{11}^{\prime } & Z_{12}^{\prime }\\ Z_{21}^{\prime } & Z_{22}^{\prime } \end{array}\right]\left[\begin{array}{c} I_{1}^{\prime }\\ I_{2}^{\prime } \end{array}\right]=\left[\begin{array}{cc} Z_{11} & Z_{13}\\ Z_{31} & Z_{33} \end{array}\right]\left[\begin{array}{c} I_{1}\\ I_{3} \end{array}\right]$$

Substituting the expressions from (8) to (11) into each component of matrix (13) yields the following:

(14) $$\left[\begin{array}{cc} Z_{11}^{\prime } & Z_{12}^{\prime }\\ Z_{21}^{\prime } & Z_{22}^{\prime } \end{array}\right]\left[\begin{array}{c} I_{1}^{\prime }\\ I_{2}^{\prime } \end{array}\right]=\left[\begin{array}{cc} -j\frac{\left(Z_{oe}+Z_{oo}\right)}{2}\cot\theta & -j\frac{\left(Z_{oe}-Z_{oo}\right)}{2}\csc\theta \\ =-j\frac{\left(Z_{oe}-Z_{oo}\right)}{2}\csc\theta & -j\frac{\left(Z_{oe}+Z_{oo}\right)}{2}\cot\theta \end{array}\right]\left[\begin{array}{c} I_{1}\\ I_{3} \end{array}\right]\;=-jZ_{oo}\cot\theta \left[\begin{array}{cc} 1 & 0\\ 0 & 1 \end{array}\right]+j\frac{\left(Z_{oe}-Z_{oo}\right)}{2}\left[\begin{array}{cc} -\cot\theta & -\csc\theta \\ -\csc\theta & -\cot\theta \end{array}\right]$$

The first term in Equation (14) can be modeled as a capacitor connected in series between Port 1 and Port 2, while the second term can be modeled as a transmission line with characteristic impedance $\frac{\left(Z_{oe}-Z_{oo}\right)}{2}$ and length θ [6].

Ultimately, Figure 4b confirms that the equivalent circuit is equivalent to a series capacitor–transmission line–series capacitor configuration. This implies that it can replace the series capacitors used in conventional interstages. Furthermore, the frequency-dependent characteristics of the transmission line demonstrate that the proposed coupled line can be used as a filter to block low-frequency signals, rather than being a simple DC block.

Strictly speaking, the implemented structure is a microstrip coupled line rather than the stripline analyzed above. Although the theoretical analysis is based on an ideal symmetric stripline structure supporting pure TEM mode, the implemented microstrip structure operates in a quasi-TEM mode and exhibits similar propagation characteristics within the frequency range of interest. Therefore, the theoretical analysis provides physical insight into the operating mechanism, while the final performance will be verified through full electromagnetic (EM) simulations.

### 2.3. Implementation of Interstage Coupled Line

**Figure 5:**
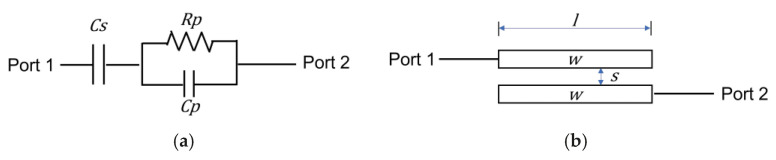
Comparison of interstage networks: (**a**) conventional RC-based structure and (**b**) proposed coupled-line structure ($C_{S}=2.2\;pF$, $C_{P}=0.2\;pF$, $R_{P}=305.8\;\Omega$, w=30 μm, s=10 μm, l=1250 μm).

To verify the characteristics of the proposed coupled-line-based interstage structure, its low-frequency behavior was compared with that of a conventional interstage network. The simulations were performed using the design kit provided by a commercial foundry. Figure 5 shows the two interstage circuit structures used for the comparative simulation. Figure 5a illustrates a conventional RC-based interstage structure consisting of a DC-blocking capacitor and an R–C parallel circuit connected in series, while Figure 5b shows the proposed coupled-line-based interstage structure.

**Figure 6:**
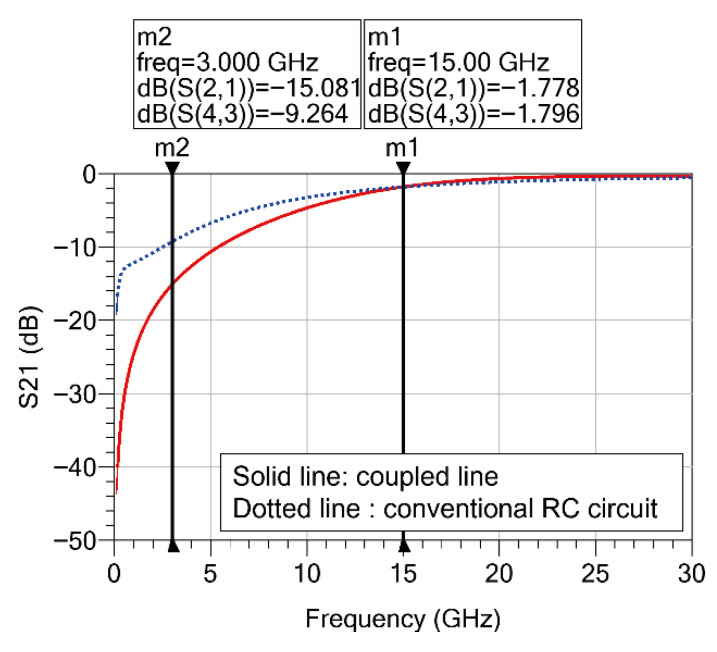
Simulated transmission characteristics of the interstage networks. The dotted line represents the conventional RC interstage circuit (Figure 5a) and the solid line represents the proposed coupled-line structure (Figure 5b).

Figure 6 shows the simulated transmission characteristics (S21) comparing the conventional RC-based interstage network and the proposed coupled-line-based interstage network. In the figure, the solid line represents the proposed coupled-line structure, while the dashed line represents the conventional RC-based interstage structure. To compare the attenuation characteristics in the low-frequency region, a marker was placed at 3 GHz. At 3 GHz, the transmission characteristic of the conventional RC-based structure is approximately −9 dB, whereas the proposed coupled-line structure exhibits a transmission level of about −15 dB, providing more than 6 dB of additional attenuation. This indicates that the coupled-line structure offers stronger gain suppression in the low-frequency region. In contrast, at 15 GHz, which corresponds to the operating frequency band, the transmission characteristics of the two structures are nearly identical. At 15 GHz, the transmission levels of the conventional RC structure and the proposed coupled-line structure are approximately −1.79 dB and −1.77 dB, respectively, showing very similar values. These results demonstrate that the proposed coupled-line structure provides additional attenuation in the low-frequency region while causing negligible degradation in power transmission within the operating band. Therefore, the proposed coupled-line-based interstage structure can serve as an effective stabilization technique for suppressing low-frequency oscillations in multistage MMIC PAs while maintaining performance in the operating frequency band.

**Figure 7:**
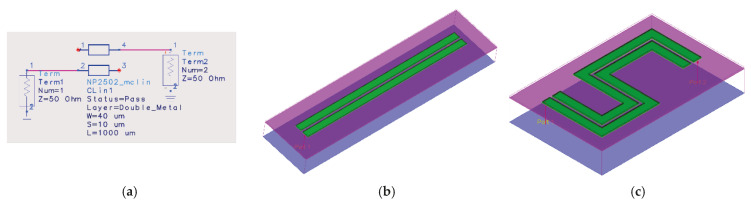
Implementation of the coupled-line structure: (**a**) circuit model used for design, (**b**) straight coupled-line EM structure, and (**c**) compact meander coupled-line structure for MMIC implementation (green color: metal1-metal2 stack layers, pink and purple color: dielectric layers).

Meanwhile, in order to apply the proposed structure to a practical MMIC circuit, it is necessary to implement the structure using physical transmission lines and verify its electromagnetic characteristics. Figure 7a shows the coupled-line model used for circuit-level simulation. This model was implemented using a coupled transmission line model, where the line width (W), spacing (S), and length (L) were defined as the main design parameters. In this design, the parameters were set to W = 40 μm, S = 10 μm, and L = 1000 μm. Figure 7b shows the three-dimensional EM model of a straight coupled-line structure implemented using the same parameters as those used in the circuit model. EM simulation was performed using this structure to verify the agreement between the circuit model and the EM model. However, the straight coupled-line structure occupies a relatively large chip area due to its long physical length. To address this issue, the transmission lines were arranged in a meander configuration, as shown in Figure 7c, so that the same electrical length could be maintained while reducing the required physical area. As a result, the horizontal dimension of the coupled-line structure was significantly reduced from 1000 μm to 570 μm by adopting the meander layout.

**Figure 8:**
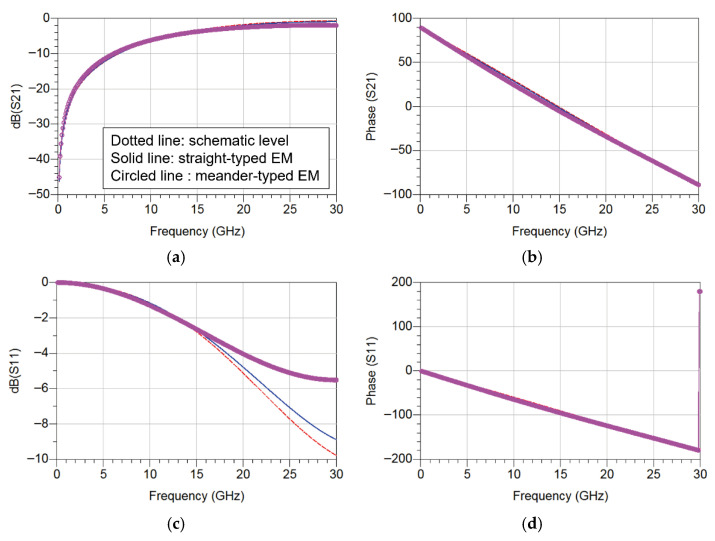
Comparison between circuit simulation and EM simulation results of the coupled-line structure ((**a**) magnitude of S21, (**b**) phase of S21, (**c**) magnitude of S11, and (**d**) phase of S11).

Figure 8 shows the comparison between the circuit simulation and EM simulation results. From the comparison of the S21 characteristics, it can be observed that the two simulation results exhibit very similar trends over the entire frequency range, indicating good agreement between the circuit model and the EM implementation of the coupled-line structure. In addition, the S11 characteristics also show similar responses in both simulations, confirming the validity of the electromagnetic implementation of the proposed structure.

EM simulations were also performed for the meander structure. The slight degradation in S11 magnitude above 15 GHz is mainly attributed to additional parasitic effects introduced by the meandered layout. Specifically, the increased line bending and proximity between adjacent segments result in additional parasitic capacitance and inductive coupling, which slightly detune the input matching network at higher frequencies. Nevertheless, they generally showed a consistent trend within the operating frequency range (up to 18 GHz). The results show that the meandered coupled-line structure maintains transmission characteristics similar to those of the coupled-line circuit model and the straight coupled-line structure, while enabling a compact layout suitable for MMIC implementation. Therefore, the size-reduced meandered coupled-line structure was adopted in the design of the Ku-band power amplifier MMIC.

## 3. Ku-Band PA MMIC Design

### 3.1. GaN HEMT Device Selection for Ku-Band PA

For the design of a high-power amplifier operating in the Ku-band, the selection of an appropriate GaN HEMT device structure and size is of critical importance. In particular, in MMIC implementations, the output power (P_out_), power-added efficiency (PAE), and stability can be significantly affected by the source grounding configuration and the gate periphery of the transistor. First, the small-signal characteristics and load–pull performance for different device sizes were analyzed, and an appropriate transistor size was finally selected based on these results. Plus, the performance differences according to the source via configuration were investigated.

**Figure 9:**
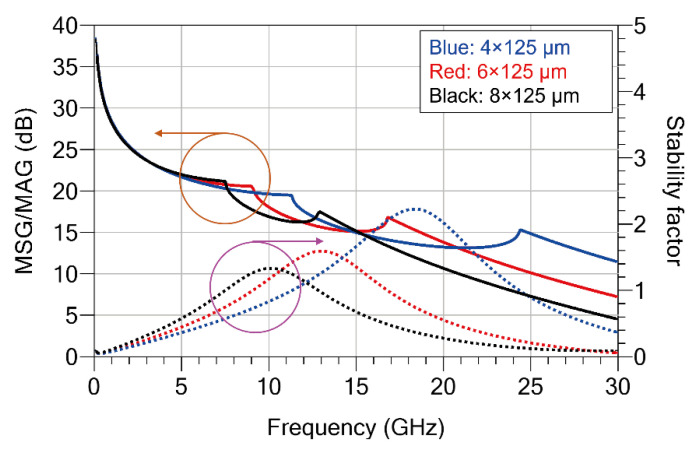
Simulated small-signal characteristics of GaN HEMT devices with different gate periphery sizes (4 × 125 μm, 6 × 125 μm, and 8 × 125 μm) at V_DD_ = 28 V, V_GG_ = −2.0 V.

To determine the appropriate gate periphery of the transistor while considering the output power and efficiency characteristics of the power amplifier, simulations were performed for three device sizes: 4 × 125 μm, 6 × 125 μm, and 8 × 125 μm. Figure 9 shows the comparison of the small-signal characteristics for each device size. The applied bias conditions were set to a drain voltage (V_DD_) of 28 V and a gate voltage (V_GG_) of −2.0 V, corresponding to Class-AB operation, in order to achieve maximum output power. The simulation results indicate that the maximum stable gain/maximum available gain (MSG/MAG) values at 15 GHz are approximately 15 dB for all three devices. However, for the 8 × 125 μm device, the gain degradation becomes more pronounced in the frequency range above 15 GHz due to the increased input and output capacitances associated with the larger gate periphery. In addition, the stability factor at 15 GHz for the 8 × 125 μm device becomes smaller than unity, indicating a higher potential for instability compared with the other device sizes.

**Figure 10:**
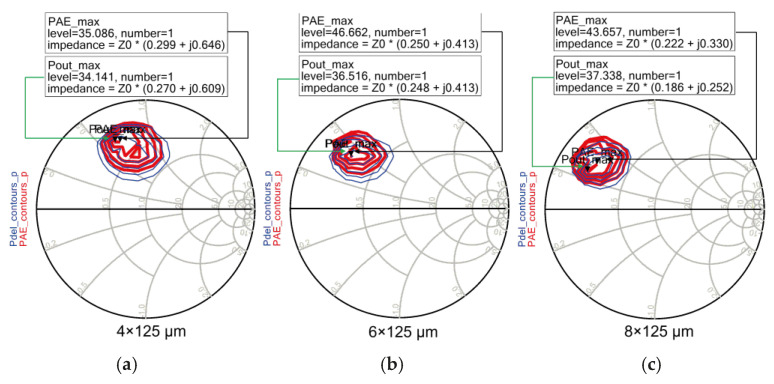
Load–pull simulation results of GaN HEMT devices with different gate periphery sizes (4 × 125 μm, 6 × 125 μm, and 8 × 125 μm), showing P_out_ (red color) and PAE (blue color) contours at 15 GHz (Pin = 30 dBm, V_DD_ = 28 V, V_GG_ = −2.0 V).

In addition, Figure 10 shows the load–pull simulation results for the three device sizes at 15 GHz. The load–pull simulation results shown in Figure 10 have been summarized in Table 1 for clearer quantitative comparison. The table includes the P_out_, gain, and power-added efficiency (PAE) for each device size. In this work, power-added efficiency (PAE) is adopted as the primary performance metric, as it accounts for both output and input power, unlike drain efficiency (DE), and therefore provides a more realistic measure of overall amplifier efficiency. By comparing the P_out_ and PAE distributions from Figure 10 and Table 1, the 6 × 125 μm device was found to exhibit the most balanced performance, achieving a maximum P_out_ of 36.5 dBm and a maximum PAE of 46.6%. For the 4 × 125 μm device, the maximum P_out_ was relatively low at 34.1 dBm. Although the 8 × 125 μm device achieved the highest P_out_ of 37.3 dBm, its maximum PAE was 43.6%, which is lower than that of the 6 × 125 μm device. In addition, the optimum load impedance (Z_opt_) of the 8 × 125 μm device was relatively smaller, which increases the complexity of the output matching network and limits the effective improvement in output power. Therefore, considering the P_out_, PAE, and gain characteristics in the Ku-band, the 6 × 125 μm GaN HEMT device was finally selected for the design of the proposed PA.

**Figure 11:**
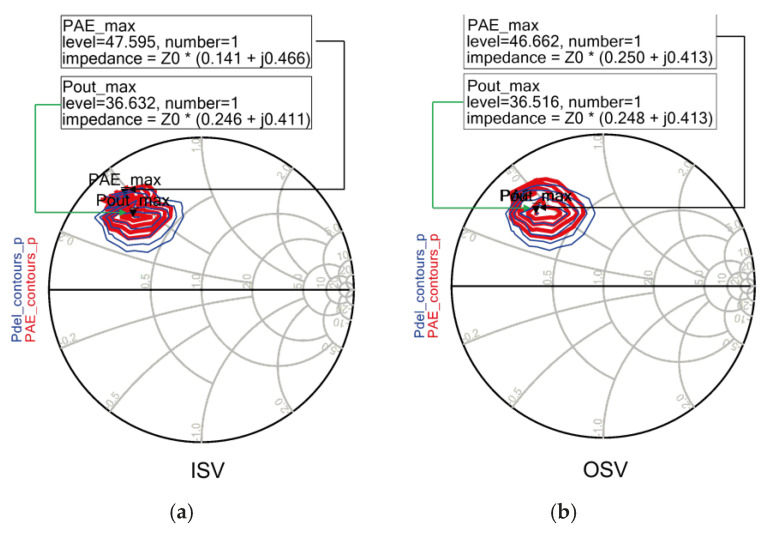
Load–pull simulation comparison between (**a**) ISV and (**b**) OSV source via structures at 15 GHz (Pin = 30 dBm, device size = 6 × 125 μm, V_DD_ = 28 V, V_GG_ = −2.0 V).

Next, to investigate the effect of the source grounding configuration, load–pull simulations were performed for two different source via structures: the inner source via (ISV) structure and the outer source via (OSV) structure. Figure 11 compares the P_out_ and PAE load–pull contours of the two structures on the Smith chart. The simulation results show that the Z_opt_ regions and PAE distributions are very similar for both structures. In addition, no significant difference in maximum P_out_ and PAE was observed between the two configurations. These results indicate that the impact of the source via location on the device performance is limited in the Ku-band operating region. Therefore, considering the ease of layout implementation and greater flexibility in MMIC placement, the OSV structure was adopted in this work.

### 3.2. Two-Stage Ku-Band Power Amplifier Design

**Figure 12:**
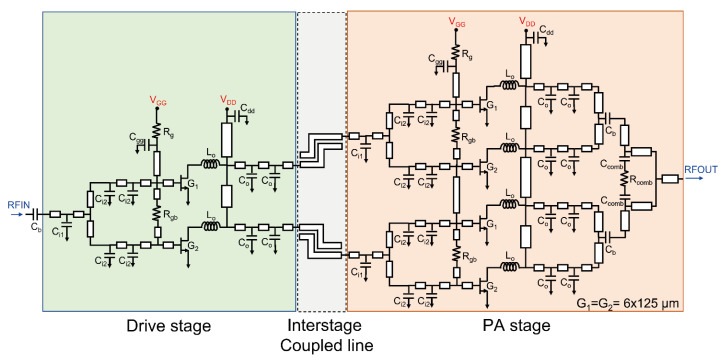
Complete circuit schematic of the proposed Ku-band two-stage GaN HEMT power amplifier MMIC.

Based on the GaN HEMT device selection and the interstage stabilization technique analyzed in the previous sections, a high-power two-stage PA MMIC for Ku-band radar applications was designed. Figure 12 shows the overall schematic of the proposed two-stage power amplifier. The overall circuit consists of a driver stage and a PA stage to simultaneously achieve high gain and high output power. Optimized matching networks were applied at each stage to ensure proper impedance matching and stable operation.

The input matching network of the driver stage was designed to distribute the RF input signal evenly to two transistors. The input matching network consists of relatively small capacitors (C_i1_ and C_i2_) together with series and inductive lines, enabling stable input matching characteristics in the Ku-band. The driver stage was designed and tuned based on the unit power cell of the output PA stage in order to provide sufficient drive capability to fully saturate the following stage.

The main PA stage, which determines the final output power, adopts a parallel configuration of two-unit power cells to achieve the target output power of more than 10 W. Each unit power cell is implemented using a 6 × 125 μm GaN HEMT device. The parasitic components of the output matching network were carefully controlled to minimize power-combining loss and maximize the achievable output power density.

All matching networks were designed using a distributed matching structure to ensure sufficient bandwidth [9]. To prevent low-frequency oscillations, series resistors (R_gb_ and R_g_) were inserted in the gate bias networks. In addition, the width of the drain bias line at the output stage was increased to accommodate a maximum current density higher than the expected operating current.

A key feature of the proposed design is the application of the meander-type coupled-line structure proposed in this work at the interstage between the driver and PA stages. Unlike conventional stabilization circuits using lumped elements, the proposed structure employs a planar transmission-line configuration, which reduces sensitivity to process variations while allowing efficient implementation within a limited chip area. As theoretically discussed in Section 2, the proposed coupled-line structure provides high attenuation in the low-frequency region while maintaining minimal insertion loss within the operating band. This characteristic effectively suppresses low-frequency oscillation, which is a common issue in multistage amplifiers.

Furthermore, by adopting a compact meander layout, the proposed structure enables efficient chip-area utilization while achieving accurate complex impedance matching between the driver and output stages. Through this systematic design approach, high power gain and stable output characteristics were achieved across the Ku-band.

**Figure 13:**
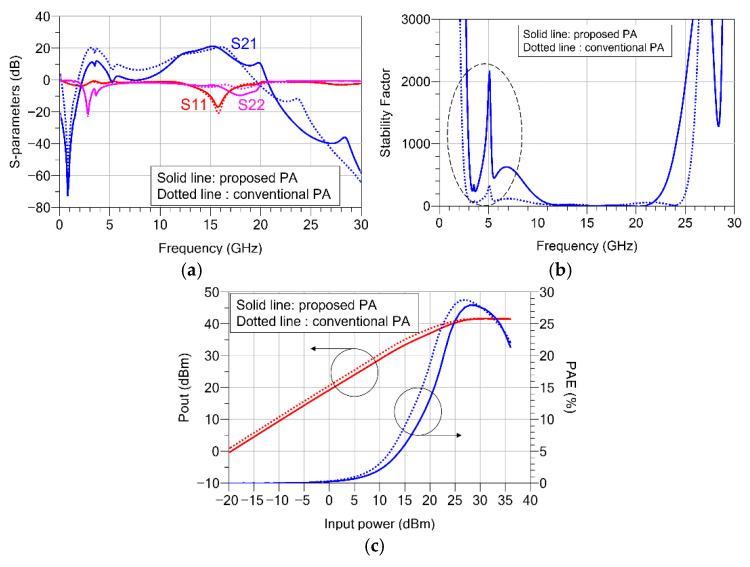
Comparison of simulated results between the conventional PA and the proposed PA with the meander-type coupled line: (**a**) small-signal S-parameters; (**b**) stability factor; (**c**) large-signal performance (Pout and PAE) versus input power at 16 GHz.

Figure 13 compares the simulation results of the proposed two-stage PA using the coupled-line interstage network with those of a conventional PA employing a typical matching structure. Specifically, the circuit-level simulations were performed using Keysight ADS 2023. First, the small-signal characteristics were examined. As shown in Figure 13a, both structures exhibit similar gain characteristics of more than 20 dB within the operating Ku-band (13.5–16 GHz). However, a clear difference appears in the low-frequency region below 5 GHz. In the conventional structure, an unnecessarily high gain is observed in the low-frequency region, which increases the risk of oscillation. In contrast, in the proposed design using the coupled-line interstage structure, the low-frequency gain is effectively suppressed to below 10 dB. To further highlight the improvement in stability at low frequencies, the stability factors were simulated and compared. As shown in Figure 13b, it can be seen that the proposed structure exhibits better stability below 10 GHz. This result indicates that the low-pass filtering characteristic of the coupled-line structure analyzed in Section 2 remains effective in the multistage amplifier design, thereby significantly improving the overall stability of the circuit.

In addition, large-signal simulations were performed at 16 GHz to verify the impact of the proposed structure on the power performance within the operating band. Figure 13c shows the comparison of P_out_ and PAE as a function of input power. The simulation results demonstrate that both the proposed PA and the conventional PA exhibit nearly identical saturated output power (P_sat_) and PAE characteristics. This confirms that the proposed meander-type coupled-line structure effectively suppresses low-frequency instability while introducing minimal additional loss in the matching network, thereby maintaining the desired high-output-power and high-efficiency performance.

## 4. Fabrication and Measurement

**Figure 14:**
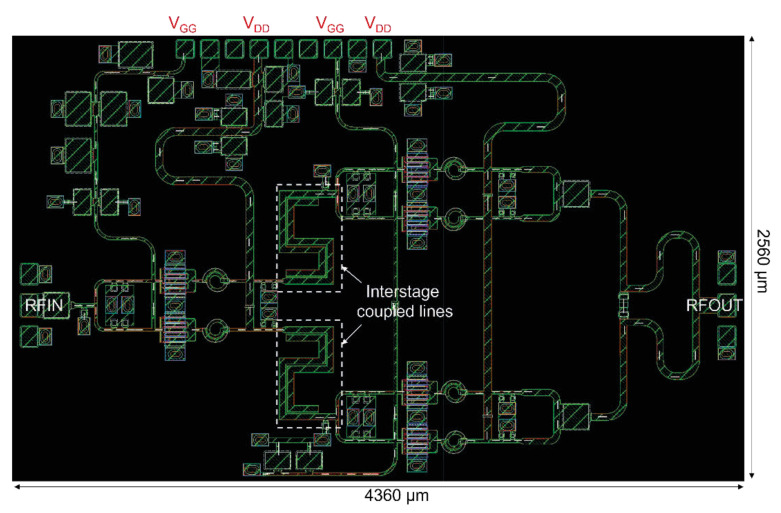
Layout of the fabricated Ku-band two-stage GaN HEMT power amplifier MMIC (chip size: 4360 × 2560 μm^2^).

The proposed Ku-band two-stage PA was fabricated using the 0.25 μm GaN-on-SiC MMIC process (NP25-00) provided by WIN semiconductor. Figure 14 shows the layout of the proposed PA MMIC. The chip size is 4360 × 2560 μm^2^. In the layout design, a symmetrical structure was adopted to minimize phase imbalance between the transistors in each stage. The RF input and output pads were placed at the left and right edges of the chip to facilitate on-wafer RF measurements.

In particular, all gate and drain bias pads were concentrated on the top side of the layout. This one-sided bias layout was intentionally adopted to facilitate future power scaling through parallel combination or module integration of multiple MMICs. By arranging the bias pads in a single direction, interference between wire bonding and external bias lines can be minimized while enabling more efficient use of the mounting area. This configuration is therefore advantageous for future system-level scalability.

As indicated by the white dashed line in the figure, a meander-type coupled-line interstage network was placed between the driver stage and the output stage to maximize layout efficiency. Compared with a straight coupled-line structure, the meander configuration significantly reduces the occupied area while preserving the intended low-frequency gain suppression and stability enhancement achieved in the design stage. The output stage employs a compact Wilkinson power combiner to combine the power from the parallel unit cells.

**Figure 15:**
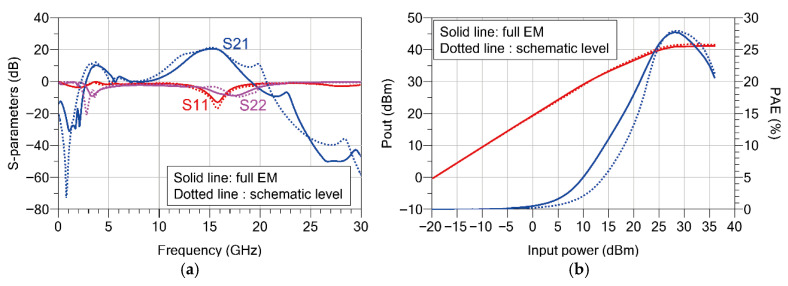
Comparison between schematic-level and full EM simulation results using ADS momentum: (**a**) small-signal S-parameters; (**b**) large-signal performance (P_out_ and PAE) at 16 GHz.

Prior to fabrication, a 2.5D full electromagnetic (EM) simulation using ADS 2023 Momentum was performed to accurately evaluate the impact of complex metal interconnections and parasitic elements in the layout. Figure 15 shows a comparison between the circuit-level (schematic) simulation results and the full EM simulation results. The EM-simulated small-signal gain (S21) was 20.5 dB at 15.2 GHz and remained above 17 dB across the 13.5–16 GHz band. The input return loss (S11) was −12.9 dB at 15.8 GHz and was better than −10 dB from 15.2 to 16.2 GHz. The output return loss (S22) was −7.7 dB at 16 GHz and remained better than −3.2 dB over the 13.5–16 GHz frequency range.

From the small-signal comparison shown in Figure 15a, it can be observed that the bandwidth in the full EM simulation becomes slightly narrower than that of the circuit-level simulation due to the parasitic inductances and capacitances introduced by the layout. However, the center frequency, which is a key design target, remains stable without a noticeable shift. In addition, a high gain of more than 20 dB and similar input/output return loss characteristics are maintained within the operating band. These results indicate that the proposed meander-type coupled-line interstage network and the matching circuits operate as intended even in the practical layout environment.

Furthermore, Figure 15b shows the large-signal simulation results at 16 GHz. At an input power of 28 dBm, the PAE was approximately 28%, and the P_sat_ was 41 dBm. Even after the full EM analysis, the P_sat_ and PAE characteristics remain in good agreement with those obtained from the circuit-level simulation. These results demonstrate that the layout design effectively suppresses performance degradation caused by parasitic elements and suggest that the fabricated MMIC is expected to exhibit performance comparable to the simulation results.

**Figure 16:**
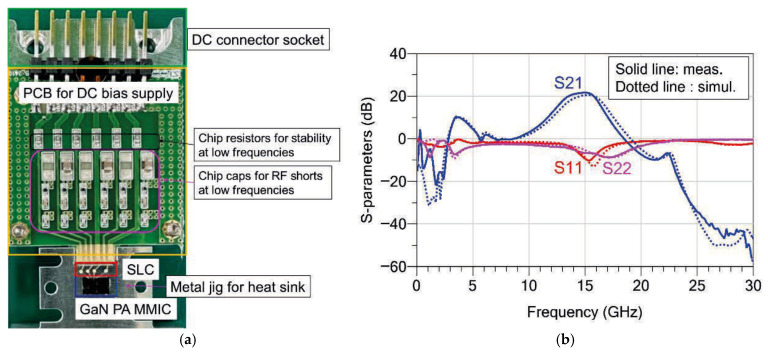
Measurement of the proposed GaN PA MMIC: (**a**) photograph of the PA MMIC module featuring the DC bias PCB, SLCs, and metal jig; (**b**) measurement result of small-signal S-parameters.

To accurately verify the RF performance of the fabricated GaN PA MMIC, a dedicated measurement module was prepared. Figure 16a shows a photograph of the measurement module for the fabricated MMIC. A separate PCB circuit was designed to supply the DC bias. To suppress potential low-frequency oscillations, a decoupling network consisting of resistors and large capacitors in the nF and tens-of-μF range was placed at the gate bias line. In particular, single-layer capacitors (SLCs) with capacitance values of several tens of pF were located close to the MMIC pads and connected through wire bonding to effectively suppress high-frequency noise [10].

For the RF input and output signals, an on-wafer probing method was adopted to evaluate the intrinsic performance of the fabricated chip. In addition, since the proposed PA targets a high output power of approximately 13 W, the entire module was mounted on a metal heat-sink jig to efficiently dissipate the generated heat and minimize performance degradation caused by thermal effects.

Figure 16b compares the simulated and measured small-signal S-parameters of the fabricated two-stage PA. The measurement results show that the input matching is slightly shifted toward lower frequencies compared with the simulation. Nevertheless, the measured results exhibit a trend very similar to the simulated characteristics within the operating frequency range of 13.5–16 GHz. Table 2 summarizes the measured S-parameters of the proposed GaN PA MMIC at 13.5, 15, and 16 GHz.

From Figure 16b and Table 2, The measured S21 was 21.6 dB at 15.0 GHz and remained above 18.5 dB across the 13.5–16 GHz band. The S11 was −10.2 dB at 15.3 GHz and was better than −3.3 dB from 13.5 to 16.0 GHz. The S22 was −7.2 dB at 16 GHz and remained better than −4.4 dB over the 13.5–16 GHz frequency range. Table 2 summarizes the measured S-parameters of the proposed GaN PA MMIC at 13.5, 15, and 16 GHz.

It is noteworthy that the effectiveness of the proposed meander-type coupled-line structure is clearly observed in the measured results. Consistent with the simulation results, the gain in the low-frequency region below 5 GHz is effectively suppressed. This result confirms the validity of the proposed structure for improving the stability of multistage amplifiers.

To verify the power performance of the fabricated two-stage PA MMIC, the P_out_, PAE, and power gain were measured as a function of frequency. To prevent thermal saturation during high-power operation and to emulate the practical operating conditions of radar systems, pulsed RF signals were used for the measurements. The pulse condition was set to a period of 100 ms with a duty cycle of 10%. In order to sufficiently drive the driver and output stages into saturation, an input power of 30 dBm was applied across the entire frequency band.

**Figure 17:**
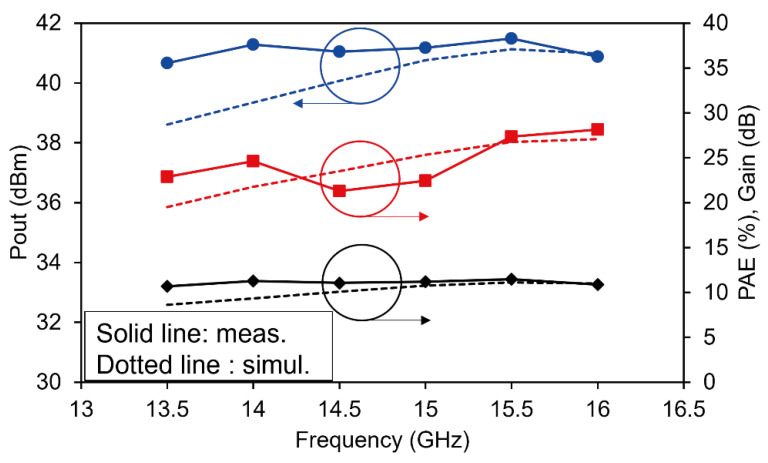
Measured pulsed large-signal performance (P_out_, PAE, and gain) versus frequency at Pin = 30 dBm (pulse conditions: 10% duty cycle, 100 ms period) (solid lines: measurement, dotted lines: simulation).

Figure 17 compares the measured results of the fabricated PA MMIC with the simulated data. For clarity, the measured results are shown with solid lines, while the simulated results are indicated by dotted lines. The measurement results show that the P_out_ ranges from 40.7 to 41.5 dBm over the frequency range of 13.5–16.5 GHz, while the PAE varies from 21.3% to 28.1%, and the power gain ranges from 10.6 to 11.1 dB. The maximum P_out_ of 41.5 dBm was obtained at 15.5 GHz, whereas the highest PAE of 28.1% was achieved at 16 GHz. The fabricated PA maintained a high P_out_ exceeding 40.5 dBm across the entire Ku-band of interest.

In particular, a maximum P_out_ of 41.5 dBm (approximately 14.1 W) was achieved at 15.5 GHz, which sufficiently satisfies the initial design target of a 13 W-class power amplifier. Compared with the simulation results (dashed lines), the overall trends of the measured output power and efficiency agree well with the simulated characteristics. Notably, the measured performance slightly exceeds the simulated results at higher frequencies. This suggests that the impedance matching of the fabricated chip is well optimized near 16 GHz. These results confirm that the proposed meander-type coupled-line interstage stabilization technique operates reliably without performance degradation even under high-power and high-efficiency conditions, and that the designed MMIC layout maintains excellent performance in a practical high-power RF environment.

The performance of the developed Ku-band two-stage GaN HEMT PA MMIC in this work is summarized in Table 3 and compared with previously reported GaN HEMT PA MMICs operating in similar frequency ranges. For a fair comparison, representative 10–20 W-class GaN HEMT PA MMICs reported in the literature were selected. As shown in Table 3, one of the main advantages of this work is the achievement of a high power density with respect to the total transistor gate width. The unit power cells used in the final stage efficiently combine the output power within a compact layout, enabling stable generation of more than 13 W of output power.

More importantly, the key contribution of this work lies in achieving a more robust and simplified design through the proposed meander-type coupled-line interstage structure. In conventional multistage high-power amplifier designs, dedicated RC stabilization networks or complex feedback circuits are typically inserted at each interstage or in front of individual transistors to suppress low-frequency oscillations. In contrast, the proposed design successfully achieves a high-power PA MMIC exceeding 10 W without oscillation using a single fabrication attempt (first-pass design), without the need for additional dedicated RC stabilization networks in the signal path. It should be noted that only minimal resistive elements are employed in the gate bias network for bias stability, rather than for primary interstage stabilization.

It should be noted that the measured input and output matching characteristics are relatively poor within the operating band. This is mainly due to the design trade-off prioritizing high output power and efficiency, as achieving optimal matching simultaneously with maximum power performance is inherently challenging in high-power GaN MMIC PAs. In particular, the use of parallel power combining structures tends to complicate impedance matching, resulting in slightly degraded S-parameter performance. Nevertheless, the achieved performance is sufficient for practical applications, as demonstrated by the stable large-signal operation and high output power. The successful first-pass implementation indicates that the proposed stabilization structure enhances design robustness by reducing sensitivity to low-frequency instability and minimizing the need for iterative tuning.

Consequently, the proposed design approach enables stable operation and compact implementation in high-power PA MMICs for radar sensor applications.

## 5. Conclusions

In this work, a 13 W-class high-power two-stage GaN PA MMIC for Ku-band radar and satellite sensor systems is presented. By applying the proposed meander-type coupled-line structure in the interstage network, low-frequency oscillations were effectively suppressed without employing complex RC stabilization circuits, thereby simplifying the overall design. The fabricated MMIC was implemented using a 0.25 μm GaN-on-SiC process. The measurement results demonstrated a maximum output power of 41.5 dBm (14.1 W) at 15.5 GHz and a peak PAE of 28.1% at 16 GHz within the Ku-band. Notably, the proposed PA achieved stable high-power operation without oscillation in a first-pass design while maintaining excellent power density. In addition, the one-sided bias layout adopted in this work provides advantages for practical module integration and system scalability. In future work, the scalability of the proposed structure to multistage or higher power amplifiers will be investigated, where stability issues become more critical. Furthermore, a systematic analysis of robustness ag统一ainst process variations and geometric tolerances, such as Monte Carlo or corner analysis, will be conducted to quantitatively assess design reliability.

## Figures and Tables

**Table 1:** Maximum P_out_, gain, and maximum PAE of GaN HEMT devices with different gate periphery sizes (4 × 125 μm, 6 × 125 μm, and 8 × 125 μm) at 15 GHz (Pin = 30 dBm, V_DD_ = 28 V, V_GG_ = −2.0 V).

	4 × 125 μm	6 × 125 μm	8 × 125 μm
Max. P_out_ (dBm)	34.14	36.52	37.34
Gain (dB)	4.14	6.52	7.34
Max. PAE (%)	35.09	46.60	43.66

**Table 2:** Measured S-parameters of the proposed GaN PA MMIC at 13.5, 15, and 16 GHz.

	13.5 GHz	15 GHz	16 GHz
S11 (dB)	−3.35	−9.39	−7.36
S21 (dB)	19.85	21.67	18.57
S22 (dB)	−4.42	−6.63	−7.22

**Table 3:** Performance comparison of Ku-band GaN HEMT PA MMICs.

Reference	Gate Length (μm)	Frequency (GHz)	Psat (dBm)	Peak PAE (%)	Gain (dB)	Total Gate Width ^1^ (mm)	Power Density ^2^ (W/mm^2^)	Chip Size (mm^2^)
[11]	0.25	10.5–15.5	38.6–39.9	42.2	18.0–21.2 ^3^	3.0	3.3	6.5
[12]	0.1	17.3–20.2	39.5–41.0 ^3^	40	–	6.4	2.0	22.5
[13]	0.25	17–20	36–40 ^3^	32	17.0–21.5	4.2	3.1	15.8
[14]	0.15	13–17	42–44	40	>30	15.4	1.6	11.2
[15]	0.25	13.5–14.5	42.5	39.5	21–22.5 ^3^	4.8	3.7	11.6
[16]	0.15	13.5–18.0	40–41	40	30–32	5.1	2.5	5.6
[17]	0.25	13.75–14.5	43	16	20	9.6	2.1	18.4
This work	0.25	13.5–16	40.7–41.5	28.1	18.6–21.6	3.0	4.7	11.2

^1^ The total gate width of the main PA stage. ^2^ Calculated as Psat/total gate width. ^3^ The data were estimated from the graphs.

## Data Availability

The original contributions presented in this study are included in the article. Further inquiries can be directed to the corresponding author.

## References

[1] ShareTechnote Communication Technology. Satellite Communication. https://www.sharetechnote.com/html/Communication_Satellite.html.

[2] Wang Y., Wang L., Wang H., Wang Y., Shu Q. (2025). Robust Cooperative Beamforming Design for Multiple Wideband LEO Satellite Networks. IEEE Access.

[3] Kim J. (2024). A Review of Ku-Band GaN HEMT Power Amplifiers Development. Micromachines.

[4] Jang Y., Choe W., Kim M., Lee Y., Jeong J. (2024). Compact GaN HEMT Power Amplifier MMIC Delivering over 40 W for Ku-Band Applications. IEEE Access.

[5] Torii T., Imai S., Maehara H., Miyashita M., Kunii T., Morimoto T., Inoue A., Ohta A., Katayama H., Yunoue N. (2016). 60% PAE, 30 W X-band and 33% PAE, 100 W Ku-band PAs Utilizing 0.15 μm GaN HEMT Technology. Proceedings of the 46th European Microwave Conference (EuMC).

[6] Pozar D. (2012). Microwave Engineering.

[7] Tripathi V.K. (1975). Asymmetric Coupled Transmission Lines in an Inhomogeneous Medium. IEEE Trans. Microw. Theory Tech..

[8] Kim J. (2026). Ultra-Wideband Asymmetric Impedance Transformer Design for High-Power Amplifier. J. Electromagn. Eng. Sci..

[9] Kim J., Choi K., Lee S., Park H., Kwon Y. (2016). 6–18 GHz Reactive Matched GaN MMIC Power Amplifiers with Distributed L-C Load Matching. J. Electromagn. Eng. Sci..

[10] Kim J., Han S., Kim B.-B., Lee M.-K., Lee B.-H. (2024). Millimeter-Wave GaN High-Power Amplifier MMIC Design Guideline Considering a Source Via Effect. Electronics.

[11] Zhang J., Nie L., Chen Y., Ren J., Ma S. (2022). A 6.5-mm^2^ 10.5–to-15.5–GHz Differential GaN PA With Coupled-Line-Based Matching Networks Achieving 10-W Peak Psat and 42% PAE. IEEE Trans. Circuits Syst. II Express Briefs.

[12] Giofrè R., Colantonio P., Costanzo F., Vitobello F., Lopez M., Cabria L. (2021). A 17.3–20.2 GHz GaN-Si MMIC Balanced HPA for Very High Throughput Satellites. IEEE Microw. Wirel. Compon. Lett..

[13] Friesicke C., Feuerschütz P., Quay R., Ambacher O., Jacob A.F. (2016). A 40 dBm AlGaN/GaN HEMT Power Amplifier MMIC for SatCom Applications at K-Band. Proceedings of the IEEE MTT-S International Microwave Symposium (IMS).

[14] Liu Y., Xiao Z., Zhu S., Wanq H., Mao S., Wu Q., Xu R., Yan B., Xu Y. A Broadband 20W GaN High Power Amplifier for Ku-band Satellite Communication. Proceedings of the 2022 IEEE International Conference on Integrated Circuits, Technologies and Applications (ICTA).

[15] Noh Y.S., Choi Y.H., Yom I. (2014). Ku-Band GaN HPA MMIC with High-Power and High-PAE Performances. Electron. Lett..

[16] Hongqi T., Jiawen W., Yi W., Dongdong M., Hanzhang C., Wen W., Tongde H. (2021). High-Power Ka/Ku Dual-Wideband GaN Power Amplifier With High Input Isolation and Transformer-Combined Load Design. IEEE Microw. Wirel. Compon. Lett..

[17] Kanaya K., Sato K., Koyanagi M., Koyama H., Tsujioka K., Ohta A. A Ku-band 20 W GaN-MMIC amplifier with built-in linearizer. Proceedings of the 2014 IEEE MTT-S International Microwave Symposium (IMS 2014).

